# 2-Hydroxy­benzyl alcohol–phenanthroline (1/1)

**DOI:** 10.1107/S1600536809044699

**Published:** 2009-10-31

**Authors:** Cuong Quoc Ton, Michael Bolte

**Affiliations:** a Institut für Organische Chemie der Goethe-Universität Frankfurt, Max-von-Laue-Strasse 7, D-60438 Frankfurt am Main, Germany; b Institut für Anorganische Chemie der Goethe-Universität Frankfurt, Max-von-Laue-Strasse 7, D-60438 Frankfurt am Main, Germany

## Abstract

Crystals of the title compound, C_12_H_8_N_2_·C_7_H_8_O_2_, were obtained during cocrystallization experiments of a compound with two hydrogen-bond donors (2-hydroxy­benzyl alcohol) with another compound containing two hydrogen-bond acceptors (phenanthroline). Unexpectedly, the two mol­ecules do not form dimers with two O—H⋯N hydrogen bonds connecting the two mol­ecules. However, one of the hydr­oxy groups forms a bifurcated hydrogen bond to both phenanthroline N atoms, whereas the other hydr­oxy group forms an O—H⋯O hydrogen bond to a symmetry-equivalent 2-hydroxy­benzyl alcohol mol­ecule. In addition, the crystal packing is stabilized by π–π inter­actions between the two phenanthroline ring systems, with a centroid–centroid distance of 3.570  Å.

## Related literature

For co-crystallization experiments, see: Ton & Bolte (2005 ▶); Tutughamiarso *et al.* (2009 ▶).
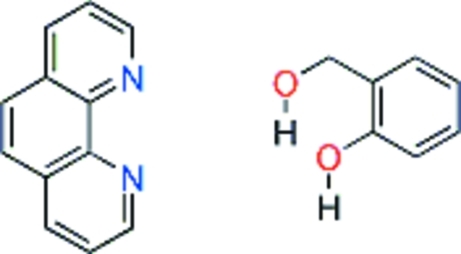


## Experimental

### 

#### Crystal data

C_12_H_8_N_2_·C_7_H_8_O_2_
*M* *_r_* = 304.34
Monoclinic, 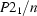

*a* = 7.264 (1) Å
*b* = 20.256 (3) Å
*c* = 11.082 (2) Å
β = 109.13 (3)°
*V* = 1540.6 (4) Å^3^
*Z* = 4
Mo *K*α radiation
μ = 0.09 mm^−1^
*T* = 173 K
0.60 × 0.50 × 0.30 mm

#### Data collection

Stoe IPDS II two-circle diffractometer
Absorption correction: none
20425 measured reflections
2885 independent reflections
2518 reflections with *I* > 2σ(*I*)
*R*_int_ = 0.036

#### Refinement

*R*[*F* ^2^ > 2σ(*F* ^2^)] = 0.038
*wR*(*F* ^2^) = 0.099
*S* = 1.06
2885 reflections
208 parameters
H-atom parameters constrained
Δρ_max_ = 0.15 e Å^−3^
Δρ_min_ = −0.21 e Å^−3^

### 

Data collection: *X-AREA* (Stoe & Cie, 2001 ▶); cell refinement: *X-AREA*; data reduction: *X-AREA*; program(s) used to solve structure: *SHELXS97* (Sheldrick, 2008 ▶); program(s) used to refine structure: *SHELXL97* (Sheldrick, 2008 ▶); molecular graphics: *XP* in *SHELXTL-Plus* (Sheldrick, 2008 ▶); software used to prepare material for publication: *SHELXL97* and *PLATON* (Spek, 2009 ▶).

## Supplementary Material

Crystal structure: contains datablocks I, global. DOI: 10.1107/S1600536809044699/om2291sup1.cif

Structure factors: contains datablocks I. DOI: 10.1107/S1600536809044699/om2291Isup2.hkl

Additional supplementary materials:  crystallographic information; 3D view; checkCIF report

## Figures and Tables

**Table 1:** Hydrogen-bond geometry (Å, °)

*D*—H⋯*A*	*D*—H	H⋯*A*	*D*⋯*A*	*D*—H⋯*A*
O1—H1*O*⋯O2^i^	0.93	1.69	2.6125 (14)	168
O2—H2*O*⋯N1^ii^	0.87	2.29	3.0390 (15)	144
O2—H2*O*⋯N2^ii^	0.87	2.15	2.8663 (14)	140

Symmetry codes: (i) 
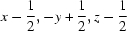
; (ii) 
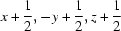
.

**Fig. 1.**
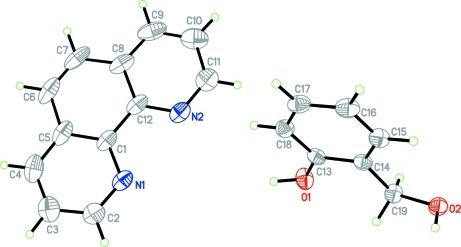
A view of the molecular structure of the title compound, with the atom-numbering scheme. Displacement ellipsoids are drawn at the 50% probability level and H atoms are shown as small spheres of arbitrary radii.

**Fig. 2.**
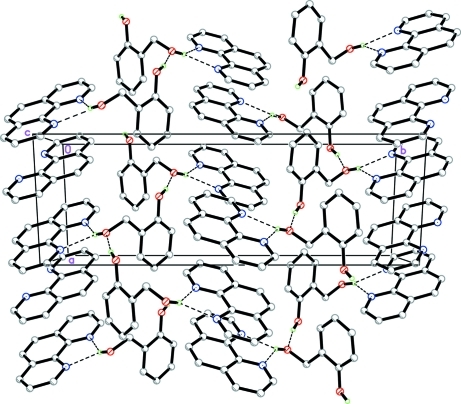
Part of the crystal structure of the title compound viewed along the c axis. Hydrogen atoms bonded to C omitted. Hydrogen bonds shown as dashed lines.

## supplementary crystallographic information

### Comment

The aim of our research is the cocrystallization of two small organic compounds in order to examine the hydrogen bonds formed between hydrogen-bond acceptors and hydrogen-bond donors (Ton & Bolte, 2005; Tutughamiarso *et al.*, 2009). In this work, we wanted to cocrystallize phenanthroline and 2-hydroxybenzyl alcohol. However, the cocrystal, we obtained, did not show the expected AA/DD pattern, *i.e.* with two O—H···N hydrogen bonds connecting the two molecules to a dimer. However, one of the hydroxy groups forms a bifurcated hydrogen bonds to both phenanthroline N atoms, whereas the other hydroxy group forms a O—H···O hydrogen bond to a symmetry equivalent 2-hydroxybenzyl alcohol molecule. In addition, the crystal packing is stabilized by π–π interactions between two phenanthroline ring systems forming a centrosymmetric dimer with a centroid···centroid distance of 3.570 Å. The second molecule is generated by the symmetry operation 1 - *x*, -*y*, 1 - *z*.

### Experimental

The complex consisting of 1,10-phenanthroline and 2-hydroxybenzylenealcohol was obtained by to the method of isothermal vaporization. 1,10-phenanthroline and 2-hydroxybenzylenealcohol were added in an equimolar ratio (10 mmol) into a flask. Afterwards chloroform was added dropwise until the substances were completely dissolved. Then, the flask was sealed and set aside at room temperature. After two weeks crystals of the complex were obtained.

### Refinement

Hydrogen atoms were located in a difference Fourier map but those bonded to C were included in calculated positions [C—H = 0.93 - 0.99 Å] and refined as riding [*U*_iso_(H) = 1.2*U*_eq_(C)]. H atoms bonded to O were freely refined.

### Crystal data

**Table:**

C_12_H_8_N_2_·C_7_H_8_O_2_	*F*(000) = 640
*M**_r_* = 304.34	*D*_x_ = 1.312 Mg m^−^^3^
Monoclinic, *P*2_1_/*n*	Mo *K*α radiation, λ = 0.71073 Å
Hall symbol: -P 2yn	Cell parameters from 11957 reflections
*a* = 7.264 (1) Å	θ = 3.0–25.0°
*b* = 20.256 (3) Å	µ = 0.09 mm^−^^1^
*c* = 11.082 (2) Å	*T* = 173 K
β = 109.13 (3)°	Block, colourless
*V* = 1540.6 (4) Å^3^	0.60 × 0.50 × 0.30 mm
*Z* = 4	

### Data collection

**Table:**

Stoe IPDS II two-circle diffractometer	2518 reflections with *I* > 2σ(*I*)
Radiation source: fine-focus sealed tube	*R*_int_ = 0.036
graphite	θ_max_ = 25.7°, θ_min_ = 2.8°
ω scans	*h* = −8→8
20425 measured reflections	*k* = −24→24
2885 independent reflections	*l* = −13→13

### Refinement

**Table:**

Refinement on *F*^2^	Primary atom site location: structure-invariant direct methods
Least-squares matrix: full	Secondary atom site location: difference Fourier map
*R*[*F*^2^ > 2σ(*F*^2^)] = 0.038	Hydrogen site location: inferred from neighbouring sites
*wR*(*F*^2^) = 0.099	H-atom parameters constrained
*S* = 1.06	*w* = 1/[σ^2^(*F*_o_^2^) + (0.0569*P*)^2^ + 0.2526*P*] where *P* = (*F*_o_^2^ + 2*F*_c_^2^)/3
2885 reflections	(Δ/σ)_max_ = 0.001
208 parameters	Δρ_max_ = 0.15 e Å^−^^3^
0 restraints	Δρ_min_ = −0.21 e Å^−^^3^

### Special details

**Table:**

Geometry. All e.s.d.'s (except the e.s.d. in the dihedral angle between two l.s. planes) are estimated using the full covariance matrix. The cell e.s.d.'s are taken into account individually in the estimation of e.s.d.'s in distances, angles and torsion angles; correlations between e.s.d.'s in cell parameters are only used when they are defined by crystal symmetry. An approximate (isotropic) treatment of cell e.s.d.'s is used for estimating e.s.d.'s involving l.s. planes.
Refinement. Refinement of *F*^2^ against ALL reflections. The weighted *R*-factor *wR* and goodness of fit *S* are based on *F*^2^, conventional *R*-factors *R* are based on *F*, with *F* set to zero for negative *F*^2^. The threshold expression of *F*^2^ > σ(*F*^2^) is used only for calculating *R*-factors(gt) *etc*. and is not relevant to the choice of reflections for refinement. *R*-factors based on *F*^2^ are statistically about twice as large as those based on *F*, and *R*- factors based on ALL data will be even larger.

### Fractional atomic coordinates and isotropic or equivalent isotropic displacement parameters (Å^2^)

**Table:**

	*x*	*y*	*z*	*U*_iso_*/*U*_eq_	
N1	0.68091 (17)	0.08958 (5)	0.49625 (11)	0.0357 (3)	
N2	0.89787 (17)	0.03273 (5)	0.72184 (11)	0.0371 (3)	
C1	0.72912 (18)	0.02470 (6)	0.49544 (13)	0.0330 (3)	
C2	0.5740 (2)	0.11589 (7)	0.38637 (14)	0.0418 (3)	
H2	0.5403	0.1612	0.3860	0.050*	
C3	0.5071 (2)	0.08146 (8)	0.27060 (15)	0.0478 (4)	
H3	0.4303	0.1029	0.1945	0.057*	
C4	0.5553 (2)	0.01591 (8)	0.26982 (15)	0.0477 (4)	
H4	0.5114	−0.0087	0.1927	0.057*	
C5	0.6694 (2)	−0.01440 (7)	0.38328 (14)	0.0398 (3)	
C6	0.7275 (2)	−0.08270 (7)	0.38997 (17)	0.0480 (4)	
H6	0.6881	−0.1088	0.3146	0.058*	
C7	0.8361 (2)	−0.11014 (7)	0.50050 (18)	0.0497 (4)	
H7	0.8733	−0.1552	0.5017	0.060*	
C8	0.8973 (2)	−0.07275 (6)	0.61706 (15)	0.0406 (3)	
C9	1.0057 (2)	−0.10017 (7)	0.73516 (18)	0.0513 (4)	
H9	1.0437	−0.1452	0.7402	0.062*	
C10	1.0568 (2)	−0.06217 (8)	0.84299 (18)	0.0526 (4)	
H10	1.1285	−0.0803	0.9238	0.063*	
C11	1.0001 (2)	0.00436 (7)	0.83078 (15)	0.0459 (4)	
H11	1.0376	0.0308	0.9057	0.055*	
C12	0.84467 (19)	−0.00511 (6)	0.61498 (13)	0.0342 (3)	
O1	0.96385 (12)	0.19916 (5)	0.97867 (8)	0.0343 (2)	
H1O	0.9040	0.1823	0.8970	0.051*	
O2	1.25869 (12)	0.33737 (4)	1.24759 (8)	0.0299 (2)	
H2O	1.2750	0.3699	1.2006	0.045*	
C13	1.16077 (16)	0.20302 (6)	1.00360 (11)	0.0254 (3)	
C14	1.26346 (16)	0.24552 (6)	1.10244 (11)	0.0252 (3)	
C15	1.46410 (17)	0.25043 (6)	1.13083 (12)	0.0300 (3)	
H15	1.5363	0.2785	1.1984	0.036*	
C16	1.56203 (18)	0.21500 (6)	1.06219 (13)	0.0333 (3)	
H16	1.6992	0.2192	1.0829	0.040*	
C17	1.45820 (18)	0.17387 (6)	0.96403 (12)	0.0325 (3)	
H17	1.5239	0.1501	0.9164	0.039*	
C18	1.25733 (18)	0.16711 (6)	0.93473 (12)	0.0293 (3)	
H18	1.1863	0.1382	0.8682	0.035*	
C19	1.15095 (17)	0.28505 (6)	1.17128 (11)	0.0297 (3)	
H19A	1.0333	0.3037	1.1071	0.036*	
H19B	1.1069	0.2548	1.2265	0.036*	

### Atomic displacement parameters (Å^2^)

**Table:**

	*U*^11^	*U*^22^	*U*^33^	*U*^12^	*U*^13^	*U*^23^
N1	0.0415 (6)	0.0277 (5)	0.0471 (7)	−0.0037 (5)	0.0269 (5)	−0.0039 (5)
N2	0.0471 (7)	0.0279 (6)	0.0456 (7)	−0.0038 (5)	0.0277 (5)	−0.0007 (5)
C1	0.0352 (7)	0.0267 (6)	0.0486 (8)	−0.0082 (5)	0.0295 (6)	−0.0070 (5)
C2	0.0450 (8)	0.0384 (8)	0.0490 (8)	−0.0024 (6)	0.0248 (7)	0.0022 (6)
C3	0.0454 (8)	0.0580 (10)	0.0469 (8)	−0.0112 (7)	0.0244 (7)	0.0013 (7)
C4	0.0474 (8)	0.0588 (10)	0.0470 (8)	−0.0211 (7)	0.0293 (7)	−0.0152 (7)
C5	0.0415 (7)	0.0374 (7)	0.0552 (9)	−0.0164 (6)	0.0356 (7)	−0.0155 (6)
C6	0.0556 (9)	0.0368 (8)	0.0695 (11)	−0.0192 (7)	0.0449 (9)	−0.0242 (7)
C7	0.0539 (9)	0.0247 (7)	0.0892 (13)	−0.0095 (6)	0.0490 (9)	−0.0169 (7)
C8	0.0388 (7)	0.0240 (6)	0.0719 (10)	−0.0049 (5)	0.0357 (7)	−0.0035 (6)
C9	0.0459 (8)	0.0278 (7)	0.0909 (13)	0.0019 (6)	0.0371 (9)	0.0081 (8)
C10	0.0484 (9)	0.0439 (8)	0.0702 (11)	0.0004 (7)	0.0257 (8)	0.0165 (8)
C11	0.0534 (9)	0.0402 (8)	0.0509 (9)	−0.0057 (6)	0.0263 (7)	0.0027 (6)
C12	0.0365 (7)	0.0250 (6)	0.0538 (8)	−0.0066 (5)	0.0321 (6)	−0.0056 (5)
O1	0.0208 (4)	0.0510 (6)	0.0310 (5)	−0.0017 (4)	0.0083 (3)	−0.0068 (4)
O2	0.0376 (5)	0.0240 (4)	0.0281 (4)	−0.0015 (3)	0.0108 (4)	0.0035 (3)
C13	0.0224 (5)	0.0288 (6)	0.0254 (6)	0.0018 (4)	0.0084 (4)	0.0058 (5)
C14	0.0248 (6)	0.0253 (6)	0.0264 (6)	0.0022 (4)	0.0094 (5)	0.0061 (5)
C15	0.0254 (6)	0.0291 (6)	0.0349 (6)	−0.0022 (5)	0.0090 (5)	0.0024 (5)
C16	0.0232 (6)	0.0341 (7)	0.0446 (7)	0.0017 (5)	0.0138 (5)	0.0059 (6)
C17	0.0323 (6)	0.0325 (6)	0.0380 (7)	0.0079 (5)	0.0188 (5)	0.0061 (5)
C18	0.0304 (6)	0.0299 (6)	0.0283 (6)	0.0024 (5)	0.0105 (5)	0.0016 (5)
C19	0.0263 (6)	0.0326 (6)	0.0311 (6)	−0.0018 (5)	0.0104 (5)	−0.0024 (5)

### Geometric parameters (Å, °)

**Table:**

N1—C2	1.3230 (19)	C10—C11	1.403 (2)
N1—C1	1.3609 (17)	C10—H10	0.9500
N2—C11	1.3235 (19)	C11—H11	0.9500
N2—C12	1.3562 (17)	O1—C13	1.3674 (14)
C1—C5	1.4166 (19)	O1—H1O	0.9310
C1—C12	1.448 (2)	O2—C19	1.4203 (15)
C2—C3	1.400 (2)	O2—H2O	0.8714
C2—H2	0.9500	C13—C18	1.3980 (17)
C3—C4	1.374 (2)	C13—C14	1.4006 (17)
C3—H3	0.9500	C14—C15	1.3901 (17)
C4—C5	1.401 (2)	C14—C19	1.5165 (16)
C4—H4	0.9500	C15—C16	1.3979 (18)
C5—C6	1.441 (2)	C15—H15	0.9500
C6—C7	1.341 (2)	C16—C17	1.3816 (19)
C6—H6	0.9500	C16—H16	0.9500
C7—C8	1.437 (2)	C17—C18	1.3938 (18)
C7—H7	0.9500	C17—H17	0.9500
C8—C9	1.402 (2)	C18—H18	0.9500
C8—C12	1.4206 (18)	C19—H19A	0.9900
C9—C10	1.366 (3)	C19—H19B	0.9900
C9—H9	0.9500		
			
C2—N1—C1	117.24 (12)	N2—C11—C10	124.36 (15)
C11—N2—C12	117.73 (12)	N2—C11—H11	117.8
N1—C1—C5	122.70 (13)	C10—C11—H11	117.8
N1—C1—C12	118.04 (12)	N2—C12—C8	122.19 (13)
C5—C1—C12	119.26 (12)	N2—C12—C1	118.51 (11)
N1—C2—C3	124.43 (14)	C8—C12—C1	119.30 (12)
N1—C2—H2	117.8	C13—O1—H1O	110.0
C3—C2—H2	117.8	C19—O2—H2O	111.4
C4—C3—C2	118.34 (15)	O1—C13—C18	122.60 (11)
C4—C3—H3	120.8	O1—C13—C14	116.55 (10)
C2—C3—H3	120.8	C18—C13—C14	120.85 (11)
C3—C4—C5	119.67 (14)	C15—C14—C13	118.11 (11)
C3—C4—H4	120.2	C15—C14—C19	123.11 (11)
C5—C4—H4	120.2	C13—C14—C19	118.77 (10)
C4—C5—C1	117.62 (13)	C14—C15—C16	121.53 (12)
C4—C5—C6	122.97 (14)	C14—C15—H15	119.2
C1—C5—C6	119.42 (15)	C16—C15—H15	119.2
C7—C6—C5	121.31 (14)	C17—C16—C15	119.61 (11)
C7—C6—H6	119.3	C17—C16—H16	120.2
C5—C6—H6	119.3	C15—C16—H16	120.2
C6—C7—C8	121.26 (13)	C16—C17—C18	120.15 (11)
C6—C7—H7	119.4	C16—C17—H17	119.9
C8—C7—H7	119.4	C18—C17—H17	119.9
C9—C8—C12	117.59 (14)	C17—C18—C13	119.73 (12)
C9—C8—C7	122.96 (14)	C17—C18—H18	120.1
C12—C8—C7	119.44 (14)	C13—C18—H18	120.1
C10—C9—C8	120.10 (14)	O2—C19—C14	114.26 (10)
C10—C9—H9	119.9	O2—C19—H19A	108.7
C8—C9—H9	119.9	C14—C19—H19A	108.7
C9—C10—C11	118.03 (16)	O2—C19—H19B	108.7
C9—C10—H10	121.0	C14—C19—H19B	108.7
C11—C10—H10	121.0	H19A—C19—H19B	107.6
			
C2—N1—C1—C5	−0.08 (18)	C11—N2—C12—C1	178.66 (11)
C2—N1—C1—C12	−179.49 (11)	C9—C8—C12—N2	0.86 (18)
C1—N1—C2—C3	0.4 (2)	C7—C8—C12—N2	179.91 (11)
N1—C2—C3—C4	−0.2 (2)	C9—C8—C12—C1	−178.65 (11)
C2—C3—C4—C5	−0.3 (2)	C7—C8—C12—C1	0.40 (18)
C3—C4—C5—C1	0.55 (19)	N1—C1—C12—N2	0.52 (17)
C3—C4—C5—C6	−179.40 (13)	C5—C1—C12—N2	−178.92 (10)
N1—C1—C5—C4	−0.37 (18)	N1—C1—C12—C8	−179.96 (11)
C12—C1—C5—C4	179.04 (11)	C5—C1—C12—C8	0.61 (17)
N1—C1—C5—C6	179.58 (11)	O1—C13—C14—C15	179.69 (10)
C12—C1—C5—C6	−1.02 (17)	C18—C13—C14—C15	−0.63 (17)
C4—C5—C6—C7	−179.66 (13)	O1—C13—C14—C19	−1.68 (15)
C1—C5—C6—C7	0.40 (19)	C18—C13—C14—C19	178.01 (11)
C5—C6—C7—C8	0.6 (2)	C13—C14—C15—C16	0.95 (17)
C6—C7—C8—C9	177.95 (13)	C19—C14—C15—C16	−177.62 (11)
C6—C7—C8—C12	−1.0 (2)	C14—C15—C16—C17	−0.24 (19)
C12—C8—C9—C10	0.1 (2)	C15—C16—C17—C18	−0.81 (18)
C7—C8—C9—C10	−178.94 (13)	C16—C17—C18—C13	1.12 (18)
C8—C9—C10—C11	−0.9 (2)	O1—C13—C18—C17	179.27 (11)
C12—N2—C11—C10	−0.1 (2)	C14—C13—C18—C17	−0.39 (17)
C9—C10—C11—N2	1.0 (2)	C15—C14—C19—O2	12.73 (16)
C11—N2—C12—C8	−0.86 (18)	C13—C14—C19—O2	−165.84 (10)

### Hydrogen-bond geometry (Å, °)

**Table:**

*D*—H···*A*	*D*—H	H···*A*	*D*···*A*	*D*—H···*A*
O1—H1O···O2^i^	0.93	1.69	2.6125 (14)	168
O2—H2O···N1^ii^	0.87	2.29	3.0390 (15)	144
O2—H2O···N2^ii^	0.87	2.15	2.8663 (14)	140

Symmetry codes: (i) *x*−1/2, −*y*+1/2, *z*−1/2; (ii) *x*+1/2, −*y*+1/2, *z*+1/2.
